# Steroid receptor coactivator-1: The central intermediator linking multiple signals and functions in the brain and spinal cord

**DOI:** 10.1016/j.gendis.2021.06.009

**Published:** 2021-07-13

**Authors:** Zhaoyou Meng, Xiaoya Wang, Dongmei Zhang, Zhen Lan, Xiaoxia Cai, Chen Bian, Jiqiang Zhang

**Affiliations:** a Department of Neurobiology, Army Medical University, Chongqing 400038, PR China; b Department of Neurosurgery, Nanchong Central Hospital, the Second Clinical Medical College, North Sichuan Medical College, Nanchong, Sichuan 637000, PR China; c Department of Dermatology, Southwest Hospital, Army Medical University, Chongqing 400038, PR China; d School of Life Sciences, Southwest University, Chongqing 400715, PR China; e School of Psychology, Amy Medical University, Chongqing 400038, PR China

**Keywords:** Cognition, Neuropathology, Neuropsychiatry, Steroid, Steroid receptor coactivator-1, Synaptic plasticity

## Abstract

The effects of steroid hormones are believed to be mediated by their nuclear receptors (NRs). The p160 coactivator family, including steroid receptor coactivator-1 (SRC-1), 2 and 3, has been shown to physically interact with NRs to enhance their transactivational activities. Among which SRC-1 has been predominantly localized in the central nervous system including brain and spinal cord. It is not only localized in neurons but also detectable in neuroglial cells (mainly localized in the nuclei but also detectable in the extra-nuclear components). Although the expression of SRC-1 is regulated by many steroids, it is also regulated by some non-steroidal factors such as injury, sound and light. Functionally, SRC-1 has been implied in normal function such as development and ageing, learning and memory, central regulation on reproductive behaviors, motor and food intake. Pathologically, SRC-1 may play a role in the regulation of neuropsychiatric disorders (including stress, depression, anxiety, and autism spectrum disorder), metabolite homeostasis and obesity as well as tumorigenesis. Under most conditions, the related mechanisms are far from elucidation; although it may regulate spatial memory through Rictor/mTORC2-actin polymerization related synaptic plasticity. Several inhibitors and stimulator of SRC-1 have shown anti-cancer potentials, but whether these small molecules could be used to modulate ageing and central disorder related neuropathology remain unclear. Therefore, to elucidate when and how SRC-1 is turned on and off under different stimuli is very interesting and great challenge for neuroscientists.

## The p160 family

Accumulated studies from recent decades have shown that steroids, including sex hormones, glucocorticoids and thyroid hormones, play fundamental roles in the central nervous system (CNS).^1^^,^^2^ The action of steroids are believed to be mediated by their nuclear receptors (NRs), such as androgen receptor (AR), estrogen receptor (ERα and ERβ), glucocorticoid (GR), thyroid receptor (TR) and progesterone receptor (PR). These NRs belong to the steroid/thyroid/retinoid receptor superfamily,^3^ they are ligand-inducible transcription factors and their association with coactivators upon binding to DNA is necessary for efficient transcriptional activity.^4^^,^^5^ Among which the p160 steroid receptor coactivator (SRC) gene family is the most extensively studied, it contains three members, namely SRC-1, SRC-2 and SRC-3.^6^ SRC-1 (also called as NCOA1) and SRC-2 (also called as TIF2/GRIP1/NCOA2) were first identified because of their abilities to enhance the transcriptional activity of the NRs tested. While SRC-3 (also called as AIB1, or RAC3/ACTR/TRAM-1/NCOA3) was originally identified by its frequent amplification in breast and ovarian cancers and subsequently demonstrated it is homologous to SRC-1 and SRC-2. Structurally, the SRC proteins are about 160 kDa in size and share 50–55% similarity and 43–48% sequence identity. Their N-terminal contains one bHLH/Per/Ah receptor nuclear translocator (ARNT)/Sim domain that is involved in DNA binding and heterodimerization between proteins containing these motifs. The central region contains the receptor interaction domain (RID), which enables direct interaction between SRCs with NRs. The RID contains three LXXLL motifs. The C-terminal region contains two domains, namely activation domains 1 and 2 (AD-1 and AD-2), responsible for recruiting secondary coregulators to the nucleating transcriptional complex.^6^^,^^7^

The SRCs have been unevenly localized in the brain. For example, Meijer et al reported that high levels of SRC-1 but low levels of SRC-2 in the brain,^8^ Apostolakis et al reported SRC-1 and SRC-2, but not SRC-3, in the ventromedial nucleus hypothalamus (VMH).^9^ Nishihara et al reported that in the adult mouse hippocampus, higher levels of SRC-1, and lower levels of SRC-2 while weak SRC-3 were detected.^10^ Overall, these and other results^11^, ^12^, ^13^, ^14^, ^15^ demonstrated that in the brain, SRC-1 expression was the highest and the most extensive, levels of SRC-2 were moderate and levels of SRC-3 were extremely low except a higher level in pituitary cells.^15^ Interestingly, the function of brain SRCs may be compensable. For instance, when SRC-1 was knocked out, levels of SRC-2 were increased^10^; high levels of hippocampal SRC-1 and very low levels of SRC-3 were detected at postnatal day (P) 0 but at P6, levels of SRC-1 were decreased while SRC-3 were increased.^11^

## The general characteristics of SRC-1

**Figure 1:**
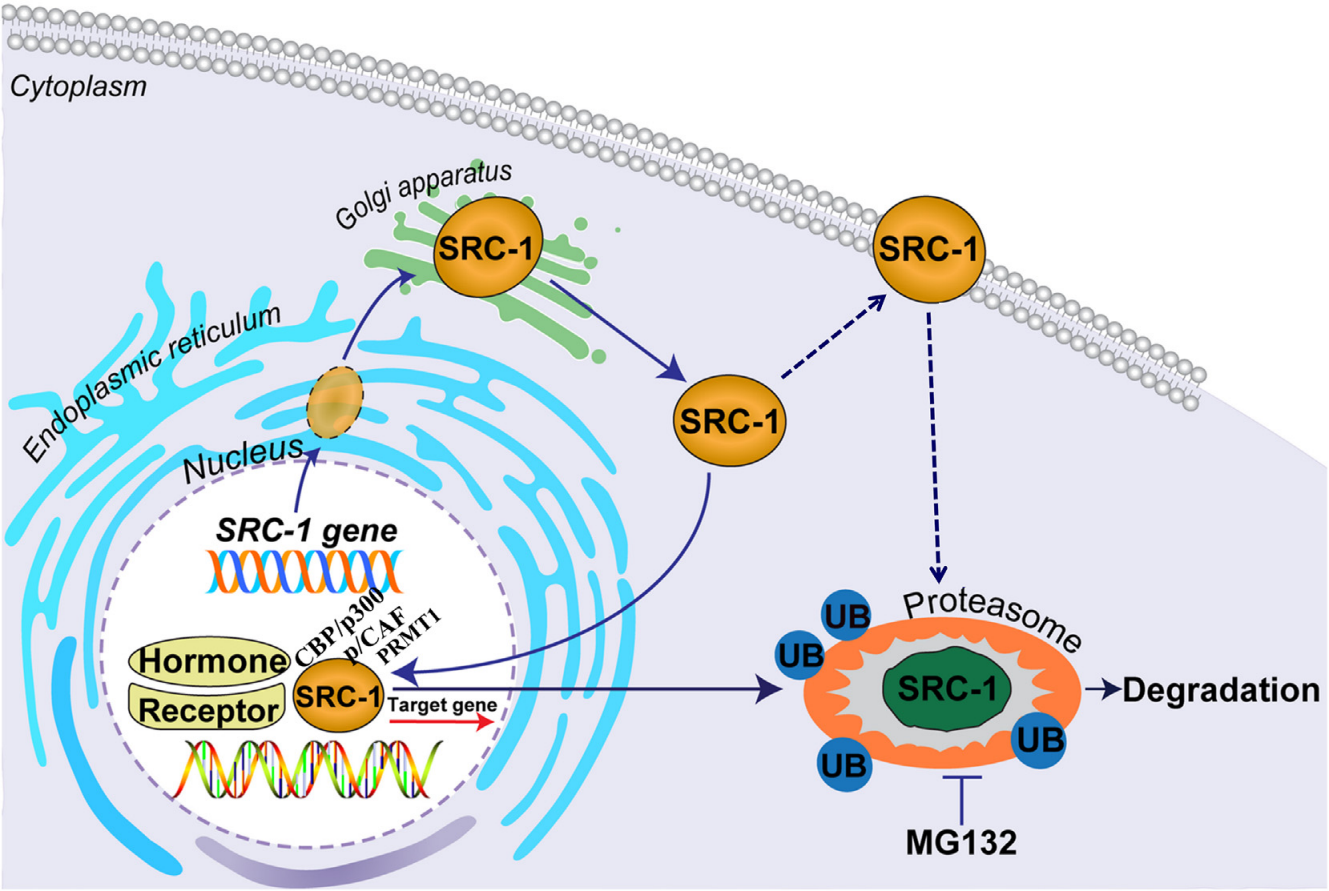
Schematic illustration of the transcriptional regulation, synthesis and degradation of SRC-1. After transcription, SRC-1 mRNA is translated into proteins in rough endoplasmic reticulum and modified in Golgi complex, the matured SRC-1 protein might be localized in the membranous structures like cell membrane and functions through the secondary messenger system; or translocated in the cell nuclei to co-activate the target genes of nuclear receptors. To regulate target gene transcription, the intra-nuclear SRC-1 transcriptional complex is formed and it contains several elements, including hormone and its nuclear receptor, SRC-1, the cAMP response element binding protein (CREB)-binding protein (CBP), p300, and the p300/CBP-associated factor (p/CAF) as well as methyltransferases including coactivator-associated arginine methyltransferase 1 and protein arginine methyltransferase 1 (PRMT1). To terminate its function, SRC-1 may need to translocate to the proteasomes and degrade by ubiquitination. The degradation of SRC-1 can be terminated by MG132, the 26S proteasome specific inhibitor. UB: ubiquitination.

SRC-1 functions to modulate ligand-dependent transactivation of several nuclear receptors, including estrogen receptor α (ERα), ERβ, androgen receptor (AR) and thyroid receptor (TR)^5^^,^^16^, ^17^, ^18^ and peroxisome proliferator-activated receptor gamma (PPARγ).^19^ The SRC-1 protein contains 19 exons and 7 LXXLL motifs (1–7), three of the motifs (3, 4, and 5) are essential for the association with nuclear receptors. It also contains two intrinsic activation domains, namely AD1 and AD2.^4^ The AD1 domain is responsible for interaction with the general transcriptional cointegrators like CREB-binding protein (CBP) and histone acetyltransferase p300 but does not interact with NRs. The AD2 domain is responsible for interaction with histone methyltransferases, coactivator-associated arginine methyltransferase 1 (CARM1) and protein arginine N-methyltransferase 1 (PRMT1).^6^ After synthesis in the cytoplasm, SRC-1 protein is imported into the nucleus, where it activates transcription and then it is translocated to the cytoplasm.^20^ The proteolysis of SRC-1 is a proteasome- and ubiquitin-mediated process that predominantly occurs in the cytoplasm,^21^ its presence was increased by inhibition of the 26S proteasome with its specific inhibitor such as MG132.^22^ Therefore, its intracellular trafficking and ubiquitinization might be a mechanism to regulate the termination of hormone action and/or the interaction with other signaling pathways in the cytoplasm as well as its degradation. Noticeably, SRC-1 immunopositive materials have also been detected in the extra-nucleus components such as cytoplasm and cell membrane,^23^, ^24^, ^25^ indicating it may also function through the second messenger pathways (Fig. 1).

## Localization of SRC-1 in the CNS

In the adult mouse brain, SRC-1 mRNA was highly expressed in the forebrain including the olfactory bulb, hippocampus, piriform cortex, amygdala, hypothalamus; it was also detected in the brainstem and cerebellum.^10^ This distribution pattern was further demonstrated by other studies in the brain of rats, Siberian hamsters^24^, ^25^, ^26^ and steroid-sensitive brain regions of songbirds (quail, canaries and zebra finch).^27^^,^^28^ In the spinal cord of adult rats, SRC-1 is abundantly expressed in the lumbar motoneurons of the spinal nucleus of the bulbocavernosus (SNB, which responds to androgens stimuli with an increased soma size), the androgen-sensitive dorsolateral nucleus, and the androgen-insensitive retrodorsolateral nucleus.^29^ Another study showed that SRC-1 was detected in the superficial laminae of the dorsal horn and within motorneurons of lamina IX.^30^ Interestingly, expression of SRC-1 was region- and sex-dependent, higher levels were usually detected in the sexually dimorphic nuclei such as in the hippocampus, preoptic area and hypothalamus, the high voice center in both songbird and rodents.^27^^,^^28^^,^^30^^,^^31^

The subcellular localization of SRC-1 exhibits cell-type and region-specific manner. In the rat CNS, SRC-1 protein has been detected predominantly in the neurons, but they were also detected in glia cells such as astrocytes and oligodendrocytes,^32^, ^33^, ^34^ ependymal cells^35^ and Schwann cells.^36^ Additionally, SRC-1 expression has been detected in several brain tumors such as astrocytic tumors,^37^ meningiomas,^38^ which further supporting the existence of SRC-1 in the glia cells. Moreover, SRC-1 immunoreactivities were mainly detected in nuclei but they were also detected in the extra-nuclear components of normal Schwann cell line (MSC 80 cells)^23^ and in the motor-related regions of rats^24^ as well as in some fiber-like structures of mice.^25^

## Steroidal regulation of brain SRC-1

Brain SRC-1 is deeply affected by steroids. Firstly, it is positively regulated by circulating androgen, since orchidectomy decreased SRC-1 in some brain regions^39^ that could be rescued by testosterone in a dose-dependent manner^40^^,^^41^; and testosterone increased the number and volumes of SRC-1 expressing cells in the preoptic area and amygdala in the green anole lizards.^42^ Secondly, it was regulated by thyroid hormones (TH) in a development- and region-specific manner since in the rat brain, anti-thyroid treatment decreased SRC-1 mRNA in the cortex and dentate gyrus but increased it in CA3.^43^ However, in mouse cerebellum, TH treatment resulted in a decrease (61%) of SRC-1 in P14 but not adult.^44^ Additionally, it has been shown that in the late gestation fetal guinea pig, repeated maternal injection with glucocorticoid had no effect on fetal limbic and anterior pituitary SRC-1 expression^45^ but in the rat brain, SRC-1 mRNA and protein were reversibly downregulated by dexamethasone.^46^^,^^47^

Many studies have reported the effects of estrogens (especially 17β-estradiol, E2) on brain SRC-1. On one hand, ovarian E2 has been shown to play profound roles in the regulation of brain structure and function through their receptors.^2^^,^^48^^,^^49^ Brain SRC-1 was positively fluctuated with estrus cycle, showing the lowest on diestrus but a significant increase at proestrus and maintenance on estrus.^50^ It was downregulated by ovariectomy (OVX) and reversed by E2 treatment.^51^ However, this regulation may be region-specific, because some studies showed that E2 increased SRC-1 in the arcuate nucleus but not the medial preoptic area or the VMH.^14^ Additional studies showed that hippocampal SRC-1 was not affected by OVX and/or E2 treatment^52^^,^^53^ in the rats but it was transiently regulated by OVX in the mice.^40^ On the other hand, it seem that brain E2, which derived from *de novo* synthesis from aromatase (estrogen synthase) by catalyzing androgens,^49^ showed positive regulation on brain SRC-1 as evidenced by results from aromatase inhibitors.^54^, ^55^, ^56^, ^57^ However, it must be pointed out that the above evidences were indeed indirect, since letrozole functions to inhibit not only central but also peripheral E2 synthesis. Therefore, how brain E2 regulated the expression of SRC-1 needs further experiments.

Interestingly, brain SRC-1 is also regulated by several non-steroidal factors such as acute stress,^58^ sound conditioning,^59^ daylength^26^ and even by injuries,^60^ highlighting its role in signal transducer for multiple signals.

## Functional implications of SRC-1 in the CNS

Different levels of SRC-1 have been detected in many regions of the CNS, from embryonic development, postnatal, adult to aged brains, under normal and pathological conditions. The expression of brain SRC-1 changes under different treatment, suggested its potentials in many brain functions.

### Development and ageing

SRC-1 plays a crucial role in brain development. it has been shown that during pre- and post-natal development, levels of brain SRC-1 exhibited region- and time-dependent.^18^^,^^45^^,^^51^^,^^61^ For example, in the hippocampus of postnatal female rats and mice, expression of SRC-1 increased with development and the highest levels were detected at P14 or P30, then it decreased to adult levels and lasted to middle-aged^24^^,^^52^^,^^62^; additional evidence showed that ageing-induced decrease of SRC-1 were seen in the spinal cord, since in the SNB of male rats, higher SRC-1 immunopositive materials were detected in the young animals but they were significantly decreased in the aged rats.^63^

The first direct evidence showing SRC-1 is involved in brain development was from Auger and colleagues. They reported that the volume of the preoptic area (POA), which is usually three to four times larger in males than in females, was decreased by 46% after neonatal infusion of SRC-1 antisense oligodeoxynucleotides (ODNs).^5^ Similar results were seen in the POA of adult quail.^64^ Nishihara et al showed that when SRC-1 was knocked out, the number of Purkinje cells was significantly decreased.^10^ The mechanisms underlying SRC-1 regulation on CNS development might involve stem cell/precursor cell differentiation, neurogenesis and myelination. One *in vitro* study revealed that during the induced differentiation of nerve stem cells (NSCs), SRC-1 was seen preferentially in neuronal lineage cells, indicating it may be involved in the neuronal-fate-committed differentiation of the NSCs.^33^ In the cortex of adult mice, SRC-1 was expressed in oligodendrocyte progenitor but not mature oligodendrocytes, indicating SRC-1 may be involved in the differentiation and maturation of oligodendrocyte progenitor.^65^ Po is a specific myelin glycoprotein of Schwann cells with a fundamental role in the maintenance and functionality of peripheral myelin. Cavarretta et al demonstrated that in Schwann cells, dihydroprogesterone (DHP) induced increase in Po and SRC-1, and the DHP induced increase in Po was enhanced by SRC-1 overexpression but inhibited by SRC-1 deficiency.^66^ Therefore, the above results strongly suggested that SRC-1 may play a role in the myelination.

### Learning and memory

Because SRC-1 is highly expressed in the cerebral cortex, hippocampus and several other nuclei that have been related to learning and memory, it is reasonably to explore its significance in the regulation of these behaviors. Nishihara et al first found that SRC-1 null mice had significantly delayed escape latencies during the training phase in the Morris water maze test.^10^ Bian et al showed that during the 5d learning phase, hippocampus-specific SRC-1 knockdown showed significant longer escape latency than control; while in the memory test, these mice spent significantly less time in the target quadrant than control.^67^ The latest study from Chen et al showed that in the hippocampus of mice, SRC-1 knockdown impaired contextual fear memory consolidation.^68^

Several studies have addressed the mechanisms underlying of SRC-1 regulation on learning and memory. The indirect evidences showed that during the postnatal development, levels of hippocampal SRC-1 and some key synaptic proteins, such as synaptophysin, PSD-95 and GluR1, shared similar profiles in both males and females,^62^ correlation analysis showed the changes of SRC-1 was positively correlated with GluR1 of the females, PSD-95 and GluR1 of the males, respectively.^40^^,^^41^ The direct clues for SRC-1 regulation on the expression of synaptic proteins showed that when a pool of SRC-1 specific shRNAs was used to block the expression of SRC-1 in the primary hippocampal neuron culture, levels of PSD-95 were decreased significantly.^69^ Zhao et al reported intra-hippocampal infusion with RNA interference lentivirus targeting SRC-1 induced significant decrease in the expression of hippocampal PSD-95 and GluR1.^57^ Bian et al showed that when hippocampal SRC-1 was inhibited, levels of hippocampal synaptic proteins (spinophilin, GluR1 and PSD-95) and CA1 synapse density as well as postsynaptic density thickness was significantly decreased.^67^ These were further supported by Chen et al showing that knockdown of hippocampal SRC-1 regulated the expression of GluR1 and PSD-95.^68^

Moreover, SRC-1 may be involved in the formation of dendritic spine and synapse. Actin polymerization contributes to the formation and maintenance of dendritic spines and synapses.^70^ In the hippocampus, levels of Rictor, phospho-AKT ser473, Cofilin (induces actin depolymerization), Profilin-1 (induces actin polymerization), and the F-actin/G-actin ratio (the marker for spine formation) have been shown to regulate hippocampal actin polymerization and hippocampus dependent memory.^71^ The *in vitro* study revealed that in the primary cultured neurons, E2 induced changes in these factors were significantly suppressed by SRC-1 inhibition^55^; and the *in vivo* evidences showed that castration and testosterone induced changes in hippocampal p-Cofilin/Cofilin ratio were significantly reversed by hippocampal SRC-1 knockdown.^57^ Furthermore, SRC-1 knockdown or SRC-1 antagonist significantly inhibited the changes in hippocampal Rictor, p-AKT and F-actin/G-actin ratio induced by activation of estrogen receptors (ERα, ERβ and GPR30,^72^, ^73^, ^74^ indicating the potential role of ERs/SRC-1/Rictor/actin polymerization pathway in the E2 regulation of hippocampal synaptic plasticity. To this end, the recent *in vivo* studies showed that when SRC-1 was inhibited, levels of hippocampal CA1 synapse density as well as postsynaptic density thickness were significantly decreased.^67^

**Figure 2:**
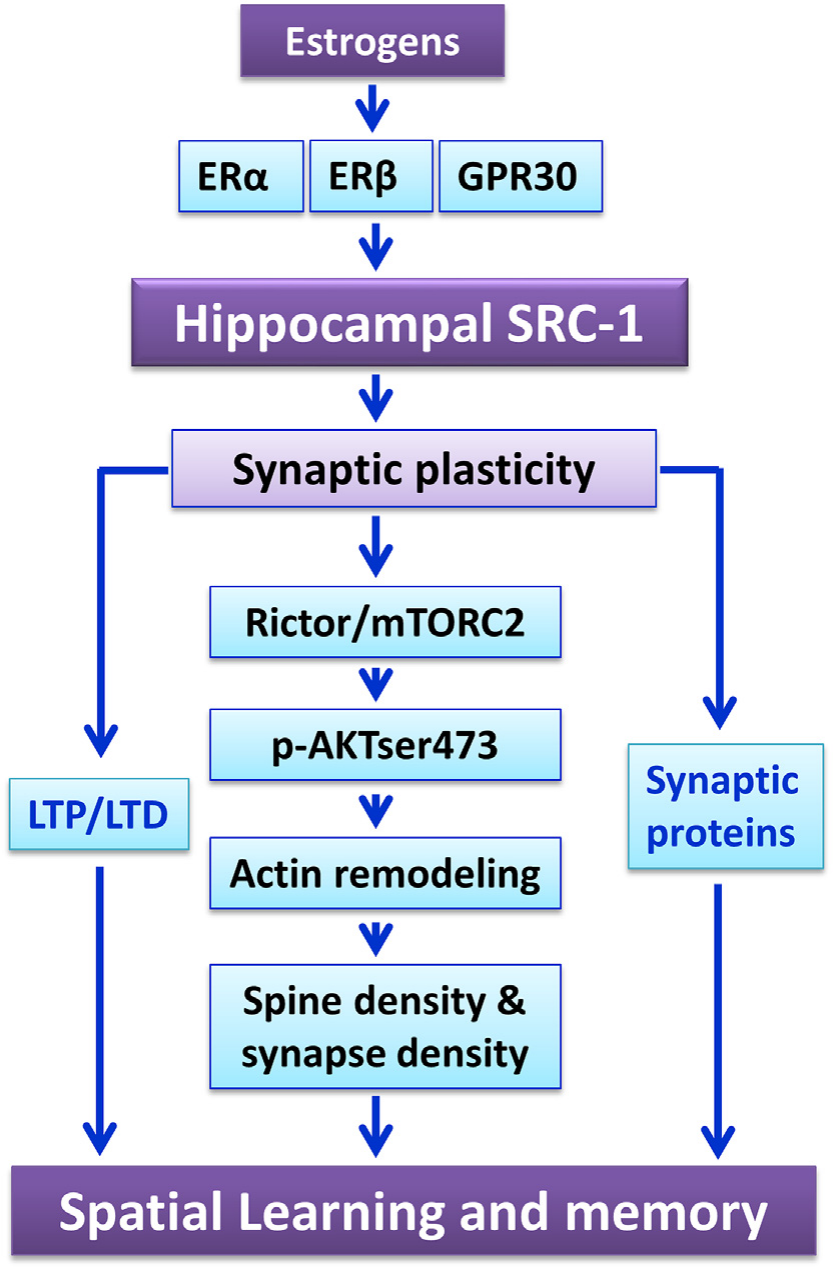
Schematic illustration of hippocampal SRC-1 in the estrogenic regulation on spatial learning and memory. In the hippocampus, levels of SRC-1 are regulated by ER (including ERα, ERβ and GPR30) agonists and antagonists. The altered expression of hippocampal SRC-1 has been found to regulate mTORC2 activity (as shown by Rictor and its downstream p-AKTser473) and actin cytoskeleton polymerization, therefore affect hippocampal spine density and synapse density. SRC-1 inhibition also impairs LTP and expression of synaptic proteins. Thus, SRC-1 plays a pivotal role in the estrogenic regulation of hippocampus-dependent spatial learning and memory through regulating several aspects of synaptic plasticity.

Taken together, current studies have shown the important role of SRC-1 in the regulation of specific synaptic proteins and actin remodeling-related proteins, dendritic spines and synapses dynamics and finally memory behavior (Fig. 2). Importantly, *in vivo* electrophysiological recording showed that long-term potentiation, the crucial marker for synaptic plasticity, was significantly impaired by SRC-1 knockdown,^67^ which also demonstrated the potential of SRC-1 in spatial memory.

### Reproductive behaviors

It has been found that neonatal infusions of SRC-1 antisense ODNs into the hypothalamus of female mice blocked the defeminizing actions of testosterone on female sexual behavior. Male and androgenized female rats neonatally infused with SRC-1 antisense ODNs displayed significantly higher levels of lordosis but did not alter the total number of mounts, intromissions, or mount latencies in male or androgenized female rats, indicating that SRC-1 is critically involved in the development of normal male reproductive behavior.^5^^,^^75^ Another study reported that in E2-treated female rats, infusion SRC-1 ODNs into the VMH significantly decreased the intensity of lordosis and reduced progestin receptor-dependent ear wiggling, hopping and darting.^76^ The medial preoptic nucleus of quail has been related to male sexual behavior,^77^ it has been found that intracerebroventricular injection of SRC-1 antisense ODNs partly blocked the activation of androgen-dependent (strutting, crowing) and estrogen-dependent (the copulatory sequence *sensu stricto*) male sexual behavior.^64^^,^^77^ Thus, these data suggest that SRC-1 play a role in the hormone-dependent sexual behaviors.

### Food intake and obesity

Hypothalamic pro-opiomelanocortin (POMC) neurons have been shown to regulate food intake and body weight and in female mice, ERα expressed in POMC neurons and steroidogenic factor-1 (SF-1) neurons mediated the anti-obesity effects of E2 administration.^78^ SRC-1 was abundantly co-localized with POMC and SF-1, its knockout significantly inhibited OVX induced body weight gain, and E2 failed to induce body weight loss in these SRC-1 null mice.^79^ Yang et al showed that SRC-1 knockout in POMC neurons induced a decrease in POMC expression, increase in food intake leading to high-fat diet-induced obesity. Since heterozygous variants in SRC-1 were found in severely obese humans, a knock-in mouse model of a loss of function human variant (SRC-1 (L1376P)) was constructed and the results showed that the food intake and body weight of these mice were significantly increased.^80^ The latest findings also revealed that in the nucleus of the solitary tract of OVX rats, SRC-1 mediated E2 induced increase in food intake and body weight.^81^ Collectively, these results indicate that targeting SRC-1 may be a useful therapeutic strategy for weight loss.

### Neuropathologies

#### Neuropsychiatric disorders

Autism spectrum disorders (ASD) are more common in males than in females. An investigation with middle frontal gyrus tissues from postmortem revealed that 34% of the subjects showed decrease in SRC-1; levels of aromatase, ERβ but not ERα were decreased by 38% and 35%, respectively. This report provided the first evidence for the possibility of E2/ERβ/SRC-1 pathway in the brain of subjects with ASD.^82^ Additionally, neuronal corticotropin-releasing hormone (CRH) released from the paraventricular nucleus (PVN) of hypothalamus and amygala has been shown to regulate depression and anxiety, respectively.^83^ SRC-1 has been detected in the PVN and amygdale,^8^^,^^24^^,^^25^^,^^84^ implying that SRC-1 may be involved in the regulation of these disorders, which has been shown to be regulated by E2.^83^ In fact, using AR-5 rat amygdala cell line, Lalmansingh et al have found that SRC-1 was necessary for the E2 induced increase in CRH expression.^83^ In the SRC-1 knockout mice, chronic stress induced upregulation of CRH expression was significantly attenuated; SRC-1 inhibition and overexpression experiments showed it was necessary for the full induction of CRH.^85^ Thus, SRC-1 was potentially involved in ASD, depression, stress and other steroid-related neuropsychiatric disorders.

#### Brain tumors

Limited studies have reported the distribution of SRC-1 in some brain tumors. SRC-1 was detected in astrocytic tumors,^37^^,^^86^^,^^87^ meningiomas,^38^ leptomeningeal specimen^38^ and pituitary microadenoma.^88^ One *in vitro* study showed that ERα agonist PPT treatment significantly increased the proliferation of astrocytic U373 and D54 cells, which could be blocked by SRC-1 inhibition.^87^ These results strongly indicated the potential role of SRC-1 in the proliferation and progression of astrocytomas and even other brain tumors. However, other studies also implied the potentials of SRC-2 especially SRC-3 in astrocytic tumors, since in this tumor high SRC-2 and SRC-3 expression was associated with worse prognosis^89^ and SRC-3 might be related to tumor differentiation.^37^ Overall, it appears that the p160 family members play important roles in the pathogenesis and progression of astrocytic tumors and might have prognostic significance.

## Conclusion and prospective

SRC-1 has been widely implicated in nuclear steroid receptor-mediated diseases during the last two decades such as breast cancer.^90^ In the CNS, SRC-1 is localized in specific regions and regulated by steroidal and non-steroidal factors; loss-of-function and gain-of–function studies have revealed it is profoundly involved in the regulation of hippocampus-dependent spatial memory, reproductive behaviors and neuropsychiatric disorders, brain tumor pathology and even required for normal motor function.^10^ Currently, when and how SRC-1 is turned on and off under different stimuli is completely unknown. The phosphorylation of SRC-1 protein may provide some clues to this end. So far seven phosphorylation sites in SRC-1 have been identified; they are serine 372, serine 395, serine 517, serine 569, serine 1033, threonine 1179, and serine 1185. All the sites contain sequences for the serine/threonine-proline-directed family of protein kinases, and two sites (serine 395 and threonine 1179) contain a sequence for the mitogen-activated protein kinase (MAPK, known to promote cell survival) family.^91^ Uncover the significance of these sites and sequences will be very beneficial for the understanding of the regulation of this coactivator.

Activation/inactivation of SRC-1 with small molecules is another interesting topic. The development of specific small molecules that can penetrate the brain blood barrier and regulation the expression and/or function of SRC-1 may provide beneficial effects against SRC-1 related central disorders. In fact, several SRC inhibitors, such as gossypol,^92^ bufalin^93^^,^^94^ and verrucarin A,^95^ or stimulator such as MCB-613^96^^,^^97^ have been tested in many cancers. Among which, bufalin significantly suppressed the growth of endometriotic lesions, Gossypol was selectively cytotoxic to cancer cells but did not affect normal cell viability, Verrucarin A was cytotoxic toward multiple types of cancer cells and MCB-613 was able to block cancer growth both *in vitro* and *in vivo*. Whether these small molecules could be effectively used to treat SRC-1 related central disorders is worthy of deep investigation.

## Conflict of interests

The authors declare that they have no conflicts of interest.

## References

[1] Lejri I., Grimm A., Eckert A. (2018). Mitochondria, estrogen and female brain aging. Front Aging Neurosci.

[2] Sarvari M., Kallo I., Hrabovszky E., Solymosi N., Liposits Z. (2017). Ovariectomy alters gene expression of the hippocampal formation in middle-aged rats. Endocrinology.

[3] Mangelsdorf D.J., Thummel C., Beato M. (1995). The nuclear receptor superfamily: the second decade. Cell.

[4] Onate S.A., Boonyaratanakornkit V., Spencer T.E. (1998). The steroid receptor coactivator-1 contains multiple receptor interacting and activation domains that cooperatively enhance the activation function 1 (AF1) and AF2 domains of steroid receptors. J Biol Chem.

[5] Auger A.P., Tetel M.J., McCarthy M.M. (2000). Steroid receptor coactivator-1 (SRC-1) mediates the development of sex-specific brain morphology and behavior. Proc Nat Acad Sci USA.

[6] Xu J., Li Q. (2003). Review of the in vivo functions of the p160 steroid receptor coactivator family. Mol Endocrinol.

[7] Szwarc M.M., Kommagani R., Lessey B.A., Lydon J.P. (2014). The p160/steroid receptor coactivator family: potent arbiters of uterine physiology and dysfunction. Biol Reprod.

[8] Meijer O.C., Steenbergen P.J., De Kloet E.R. (2000). Differential expression and regional distribution of steroid receptor coactivators SRC-1 and SRC-2 in brain and pituitary. Endocrinology.

[9] Apostolakis E.M., Ramamurphy M., Zhou D., Oñate S., O'Malley B.W. (2002). Acute disruption of select steroid receptor coactivators prevents reproductive behavior in rats and unmasks genetic adaptation in knockout mice. Mol Endocrinol.

[10] Nishihara E., Yoshida-Komiya H., Chan C.S. (2003). SRC-1 null mice exhibit moderate motor dysfunction and delayed development of cerebellar Purkinje cells. J Neurosci.

[11] Schmidt M.V., Oitzl M., Steenbergen P. (2007). Ontogeny of steroid receptor coactivators in the hippocampus and their role in regulating postnatal HPA axis function. Brain Res.

[12] Yore M.A., Im D., Webb L.K. (2010). Steroid receptor coactivator-2 expression in brain and physical associations with steroid receptors. Neuroscience.

[13] Acharya K.D., Finkelstein S.D., Bless E.P. (2015). Estradiol preferentially induces progestin receptor-A (PR-A) over PR-B in cells expressing nuclear receptor coactivators in the female mouse hypothalamus. eNeuro.

[14] Tognoni C.M., Chadwick J.G., Ackeifi C.A., Tetel M.J. (2011). Nuclear receptor coactivators are coexpressed with steroid receptors and regulated by estradiol in mouse brain. Neuroendocrinology.

[15] An B.S., Selva D.M., Hammond G.L., Rivero-Muller A., Rahman N., Leung P.C. (2006). Steroid receptor coactivator-3 is required for progesterone receptor trans-activation of target genes in response to gonadotropin-releasing hormone treatment of pituitary cells. J Biol Chem.

[16] Kamiya Y., Zhang X.Y., Ying H. (2003). Modulation by steroid receptor coactivator-1 of target-tissue responsiveness in resistance to thyroid hormone. Endocrinology.

[17] Molenda-Figueira H.A., Murphy S.D., Shea K.L. (2008). Steroid receptor coactivator-1 from brain physically interacts differentially with steroid receptor subtypes. Endocrinology.

[18] Yousefi B., Jingu H., Ohta M., Umezu M., Koibuchi N. (2005). Postnatal changes of steroid receptor coactivator-1 immunoreactivity in rat cerebellar cortex. Thyroid.

[19] Zhu Y., Qi C., Calandra C., Rao M.S., Reddy J.K. (1996). Cloning and identification of mouse steroid receptor coactivator-1 (mSRC-1), as a coactivator of peroxisome proliferator-activated receptor gamma. Gene Expr.

[20] Amazit L., Alj Y., Tyagi R.K. (2003). Subcellular localization and mechanisms of nucleocytoplasmic trafficking of steroid receptor coactivator-1. J Biol Chem.

[21] Amazit L., Roseau A., Khan J.A. (2011). Ligand-dependent degradation of SRC-1 is pivotal for progesterone receptor transcriptional activity. Mol Endocrinol.

[22] Villamar-Cruz O., Manjarrez-Marmolejo J., Alvarado R., Camacho-Arroyo I. (2006). Regulation of the content of progesterone and estrogen receptors, and their cofactors SRC-1 and SMRT by the 26S proteasome in the rat brain during the estrous cycle. Brain Res Bull.

[23] Grenier J., Trousson A., Chauchereau A., Cartaud J., Schumacher M., Massaad C. (2006). Differential recruitment of p160 coactivators by glucocorticoid receptor between Schwann cells and astrocytes. Mol Endocrinol.

[24] Zhang D., Guo Q., Bian C., Zhang J., Lin S., Su B. (2011). Alterations of steroid receptor coactivator-1 (SRC-1) immunoreactivities in specific brain regions of young and middle-aged female Sprague-Dawley rats. Brain Res.

[25] Bian C., Zhang D., Guo Q., Cai W., Zhang J. (2011). Localization and sex-difference of steroid receptor coactivator-1 immunoreactivities in the brain of adult female and male mice. Steroids.

[26] Tetel M.J., Ungar T.C., Hassan B., Bittman E.L. (2004). Photoperiodic regulation of androgen receptor and steroid receptor coactivator-1 in Siberian hamster brain. Brain Res Mol Brain Res.

[27] Charlier T.D., Balthazart J., Ball G.F. (2003). Sex differences in the distribution of the steroid receptor coactivator SRC-1 in the song control nuclei of male and female canaries. Brain Res.

[28] Duncan K.A., Jimenez P., Carruth L.L. (2011). Distribution and sexually dimorphic expression of steroid receptor coactivator-1 (SRC-1) in the zebra finch brain. Gen Comp Endocrinol.

[29] O'Bryant E.L., Jordan C.L. (2005). Expression of nuclear receptor coactivators in androgen-responsive and -unresponsive motoneurons. Horm Behav.

[30] Ranson R.N., Santer R.M., Watson A.H. (2003). SRC-1 localisation in lumbosacral spinal cord of male and female Wistar rats. Neuroreport.

[31] Xiao J., Zhang J., Zhao Y. (2017). Sex differences of steroid receptor coactivator-1 expression after spinal cord injury in mice. Neurol Res.

[32] Ogawa H., Nishi M., Kawata M. (2001). Localization of nuclear coactivators p300 and steroid receptor coactivator 1 in the rat hippocampus. Brain Res.

[33] Nishihara E., Moriya T., Shinohara K. (2007). Expression of steroid receptor coactivator-1 is elevated during neuronal differentiation of murine neural stem cells. Brain Res.

[34] Morte B., Gil-Ibáñez P., Bernal J. (2018). Regulation of gene expression by thyroid hormone in primary astrocytes: factors influencing the genomic response. Endocrinology.

[35] Iwata K., Ozawa H. (2014). Expression of glucocorticoid receptor and coactivators in ependymal cells of male rats. Acta Histochem Cytoc.

[36] Grenier J., Trousson A., Chauchereau A. (2004). Selective recruitment of p160 coactivators on glucocorticoid-regulated promoters in Schwann cells. Mol Endocrinol.

[37] Liu C., Zhang Y., Zhang K., Bian C., Zhao Y., Zhang J. (2014). Expression of estrogen receptors, androgen receptor and steroid receptor coactivator-3 is negatively correlated to the differentiation of astrocytic tumors. Cancer Epidemiol.

[38] Carroll R.S., Brown M., Zhang J., DiRenzo J., Font De Mora J., Black P.M. (2000). Expression of a subset of steroid receptor cofactors is associated with progesterone receptor expression in meningiomas. Clin Cancer Res.

[39] Bian C., Zhang K., Zhao Y., Guo Q., Cai W., Zhang J. (2014). Regional specific regulation of steroid receptor coactivator-1 immunoreactivity by orchidectomy in the brain of adult male mice. Steroids.

[40] Bian C., Zhu K., Yang L. (2012). Gonadectomy differentially regulates steroid receptor coactivator-1 and synaptic proteins in the hippocampus of adult female and male C57BL/6 mice. Synapse (N Y).

[41] Qiu L., Zhao Y., Guo Q. (2016). Dose-dependent regulation of steroid receptor coactivator-1 and steroid receptors by testosterone propionate in the hippocampus of adult male mice. J Steroid Biochem Mol Biol.

[42] Kerver H.N., Wade J. (2015). Hormonal regulation of steroid receptor coactivator-1 mRNA in the male and female green anole brain. J Neuroendocrinol.

[43] Iannacone E.A., Yan A.W., Gauger K.J., Dowling A.L., Zoeller R.T. (2002). Thyroid hormone exerts site-specific effects on SRC-1 and NCoR expression selectively in the neonatal rat brain. Mol Cell Endocrinol.

[44] Ramos H.E., Weiss R.E. (2006). Regulation of nuclear coactivator and corepressor expression in mouse cerebellum by thyroid hormone. Thyroid.

[45] Setiawan E., Owen D., McCabe L., Kostaki A., Andrews M.H., Matthews S.G. (2004). Glucocorticoids do not alter developmental expression of hippocampal or pituitary steroid receptor coactivator-1 and -2 in the late gestation fetal Guinea pig. Endocrinology.

[46] Kurihara I., Shibata H., Suzuki T. (2000). Transcriptional regulation of steroid receptor coactivator-1 (SRC-1) in glucocorticoid action. Endocr Res.

[47] Kurihara I., Shibata H., Suzuki T. (2002). Expression and regulation of nuclear receptor coactivators in glucocorticoid action. Mol Cell Endocrinol.

[48] Bastos C.P., Pereira L.M., Ferreira-Vieira T.H. (2015). Object recognition memory deficit and depressive-like behavior caused by chronic ovariectomy can be transitorialy recovered by the acute activation of hippocampal estrogen receptors. Psychoneuroendocrinology.

[49] Zhao Y., He L., Zhang Y. (2017). Estrogen receptor alpha and beta regulate actin polymerization and spatial memory through an SRC-1/mTORC2-dependent pathway in the hippocampus of female mice. J Steroid Biochem Mol Biol.

[50] Camacho-Arroyo I., Neri-Gomez T., Gonzalez-Arenas A., Guerra-Araiza C. (2005). Changes in the content of steroid receptor coactivator-1 and silencing mediator for retinoid and thyroid hormone receptors in the rat brain during the estrous cycle. J Steroid Biochem Mol Biol.

[51] Mitev Y.A., Wolf S.S., Almeida O.F., Patchev V.K. (2003). Developmental expression profiles and distinct regional estrogen responsiveness suggest a novel role for the steroid receptor coactivator SRC-1 as discriminative amplifier of estrogen signaling in the rat brain. FASEB J.

[52] Zhang D., Guo Q., Bian C., Zhang J., Cai W., Su B. (2011). Expression of steroid receptor coactivator-1 was regulated by postnatal development but not ovariectomy in the hippocampus of rats. Dev Neurosci.

[53] Bohacek J., Daniel J.M. (2009). The ability of oestradiol administration to regulate protein levels of oestrogen receptor alpha in the hippocampus and prefrontal cortex of middle-aged rats is altered following long-term ovarian hormone deprivation. J Neuroendocrinol.

[54] Bian C., Zhao Y., Guo Q., Xiong Y., Cai W., Zhang J. (2014). Aromatase inhibitor letrozole downregulates steroid receptor coactivator-1 in specific brain regions that primarily related to memory, neuroendocrine and integration. J Steroid Biochem Mol Biol.

[55] Zhao Y., Yu Y., Zhang Y. (2017). Letrozole regulates actin cytoskeleton polymerization dynamics in a SRC-1 dependent manner in the hippocampus of mice. J Steroid Biochem Mol Biol.

[56] Liu M., Xing F., Bian C. (2019). Letrozole induces worse hippocampal synaptic and dendritic changes and spatial memory impairment than ovariectomy in adult female mice. Neurosci Lett.

[57] Zhao J., Bian C., Liu M. (2018). Orchiectomy and letrozole differentially regulate synaptic plasticity and spatial memory in a manner that is mediated by SRC-1 in the hippocampus of male mice. J Steroid Biochem Mol Biol.

[58] Bousios S., Karandrea D., Kittas C., Kitraki E. (2001). Effects of gender and stress on the regulation of steroid receptor coactivator-1 expression in the rat brain and pituitary. J Steroid Biochem Mol Biol.

[59] Tahera Y., Meltser I., Johansson P., Salman H., Canlon B. (2007). Sound conditioning protects hearing by activating the hypothalamic-pituitary-adrenal axis. Neurobiol Dis.

[60] Xiao J., Zhang J., Zhao Y. (2017). Sex differences of steroid receptor coactivator-1 expression after spinal cord injury in mice. Neurol Res.

[61] Misiti S., Koibuchi N., Bei M., Farsetti A., Chin W.W. (1999). Expression of steroid receptor coactivator-1 mRNA in the developing mouse embryo: a possible role in olfactory epithelium development. Endocrinology.

[62] Bian C., Zhu K., Guo Q., Xiong Y., Cai W., Zhang J. (2012). Sex differences and synchronous development of steroid receptor coactivator-1 and synaptic proteins in the hippocampus of postnatal female and male C57BL/6 mice. Steroids.

[63] Matsumoto A. (2002). Age-related changes in nuclear receptor coactivator immunoreactivity in motoneurons of the spinal nucleus of the bulbocavernosus of male rats. Brain Res.

[64] Charlier T.D., Ball G.F., Balthazart J. (2005). Inhibition of steroid receptor coactivator-1 blocks estrogen and androgen action on male sex behavior and associated brain plasticity. J Neurosci : Offl J Soc Neurosci.

[65] Matsusue Y., Horii-Hayashi N., Kirita T., Nishi M. (2014). Distribution of corticosteroid receptors in mature oligodendrocytes and oligodendrocyte progenitors of the adult mouse brain. J Histochem Cytochem.

[66] Cavarretta I.T., Martini L., Motta M., Smith C.L., Melcangi R.C. (2004). SRC-1 is involved in the control of the gene expression of myelin protein Po. J Mol Neurosci.

[67] Bian C., Huang Y., Zhu H., Zhao Y., Zhao J., Zhang J. (2018). Steroid receptor coactivator-1 knockdown decreases synaptic plasticity and impairs spatial memory in the Hippocampus of mice. Neuroscience.

[68] Chen X., Tian Y., Zhu H., Bian C., Li M. (2020). Inhibition of steroid receptor coactivator-1 in the hippocampus impairs the consolidation and reconsolidation of contextual fear memory in mice. Life Sci.

[69] Liu M., Huangfu X., Zhao Y., Zhang D., Zhang J. (2015). Steroid receptor coactivator-1 mediates letrozole induced downregulation of postsynaptic protein PSD-95 in the hippocampus of adult female rats. J Steroid Biochem Mol Biol.

[70] Shi Y., Pontrello C.G., DeFea K.A., Reichardt L.F., Ethell I.M. (2009). Focal adhesion kinase acts downstream of EphB receptors to maintain mature dendritic spines by regulating cofilin activity. J Neurosci.

[71] Huang W., Zhu P.J., Zhang S. (2013). mTORC2 controls actin polymerization required for consolidation of long-term memory. Nat Neurosci.

[72] Zhao Y., He L., Zhang Y. (2017). Estrogen receptor alpha and beta regulate actin polymerization and spatial memory through an SRC-1/mTORC2-dependent pathway in the Hippocampus of female mice. J Steroid Biochem Mol Biol.

[73] Xing F.Z., Zhao Y.G., Zhang Y.Y. (2018). Nuclear and membrane estrogen receptor antagonists induce similar mTORC2 activation-reversible changes in synaptic protein expression and actin polymerization in the mouse hippocampus. CNS Neurosci Ther.

[74] Zhang Y.Y., Liu M.Y., Liu Z. (2019). GPR30-mediated estrogenic regulation of actin polymerization and spatial memory involves SRC-1 and PI3K-mTORC2 in the hippocampus of female mice. CNS Neurosci Ther.

[75] Molenda H.A., Griffin A.L., Auger A.P., McCarthy M.M., Tetel M.J. (2002). Nuclear receptor coactivators modulate hormone-dependent gene expression in brain and female reproductive behavior in rats. Endocrinology.

[76] Molenda-Figueira H.A., Williams C.A., Griffin A.L., Rutledge E.M., Blaustein J.D., Tetel M.J. (2006). Nuclear receptor coactivators function in estrogen receptor- and progestin receptor-dependent aspects of sexual behavior in female rats. Horm Behav.

[77] Balthazart J., Charlier T.D., Barker J.M., Yamamura T., Ball G.F. (2010). Sex steroid-induced neuroplasticity and behavioral activation in birds. Eur J Neurosci.

[78] Xu Y., Nedungadi T.P., Zhu L. (2011). Distinct hypothalamic neurons mediate estrogenic effects on energy homeostasis and reproduction. Cell Metabol.

[79] Zhu L., Yang Y., Xu P. (2013). Steroid receptor coactivator-1 mediates estrogenic actions to prevent body weight gain in female mice. Endocrinology.

[80] Yang Y., van der Klaauw A.A., Zhu L. (2019). Steroid receptor coactivator-1 modulates the function of Pomc neurons and energy homeostasis. Nat Commun.

[81] Shen L., Liu Y., Tso P. (2018). Silencing steroid receptor coactivator-1 in the nucleus of the solitary tract reduces estrogenic effects on feeding and apolipoprotein A-IV expression. J Biol Chem.

[82] Crider A., Thakkar R., Ahmed A.O., Pillai A. (2014). Dysregulation of estrogen receptor beta (ERbeta), aromatase (CYP19A1), and ER co-activators in the middle frontal gyrus of autism spectrum disorder subjects. Mol Autism.

[83] Lalmansingh A.S., Uht R.M. (2008). Estradiol regulates corticotropin-releasing hormone gene (crh) expression in a rapid and phasic manner that parallels estrogen receptor-alpha and -beta recruitment to a 3',5'-cyclic adenosine 5'-monophosphate regulatory region of the proximal crh promoter. Endocrinology.

[84] Hinton A.O., Yang Y., Quick A.P. (2016). SRC-1 regulates blood pressure and aortic stiffness in female mice. PLoS One.

[85] Lachize S., Apostolakis E.M., van der Laan S. (2009). Steroid receptor coactivator-1 is necessary for regulation of corticotropin-releasing hormone by chronic stress and glucocorticoids. Proc Nat Acad Sci USA.

[86] Hernandez-Hernandez O.T., Rodriguez-Dorantes M., Gonzalez-Arenas A., Camacho-Arroyo I. (2010). Progesterone and estradiol effects on SRC-1 and SRC-3 expression in human astrocytoma cell lines. Endocrine.

[87] Gonzalez-Arenas A., Hansberg-Pastor V., Hernandez-Hernandez O.T. (2012). Estradiol increases cell growth in human astrocytoma cell lines through ERalpha activation and its interaction with SRC-1 and SRC-3 coactivators. Biochim Biophys Acta.

[88] Usui T., Izawa S., Sano T. (2005). Clinical and molecular features of a TSH-secreting pituitary microadenoma. Pituitary.

[89] Kefalopoulou Z., Tzelepi V., Zolota V. (2012). Prognostic value of novel biomarkers in astrocytic brain tumors: nuclear receptor co-regulators AIB1, TIF2, and PELP1 are associated with high tumor grade and worse patient prognosis. J Neuro Oncol.

[90] Johnson A.B., O'Malley B.W. (2012). Steroid receptor coactivators 1, 2, and 3: critical regulators of nuclear receptor activity and steroid receptor modulator (SRM)-based cancer therapy. Mol Cell Endocrinol.

[91] Rowan B.G., Weigel N.L., O'Malley B.W. (2000). Phosphorylation of steroid receptor coactivator-1. Identification of the phosphorylation sites and phosphorylation through the mitogen-activated protein kinase pathway. J Biol Chem.

[92] Wang Y., Lonard D.M., Yu Y., Chow D.C., Palzkill T.G., O'Malley B.W. (2011). Small molecule inhibition of the steroid receptor coactivators, SRC-3 and SRC-1. Mol Endocrinol.

[93] Cho Y.J., Lee J.E., Park M.J., O'Malley B.W., Han S.J. (2018). Bufalin suppresses endometriosis progression by inducing pyroptosis and apoptosis. J Endocrinol.

[94] Wang Y., Lonard D.M., Yu Y. (2014). Bufalin is a potent small-molecule inhibitor of the steroid receptor coactivators SRC-3 and SRC-1. Cancer Res.

[95] Yan F., Yu Y., Chow D.C. (2014). Identification of verrucarin a as a potent and selective steroid receptor coactivator-3 small molecule inhibitor. PLoS One.

[96] Lonard D.M., O'Malley B.W. (2016). Molecular pathways: targeting steroid receptor coactivators in cancer. Clin Cancer Res.

[97] Wang L., Yu Y., Chow D.C. (2015). Characterization of a steroid receptor coactivator small molecule stimulator that overstimulates cancer cells and leads to cell stress and death. Cancer Cell.

